# Limitations of the dorsal skinfold window chamber model in evaluating anti-angiogenic therapy during early phase of angiogenesis

**DOI:** 10.1186/2045-824X-6-17

**Published:** 2014-08-04

**Authors:** Nikolett M Biel, Jennifer A Lee, Brian S Sorg, Dietmar W Siemann

**Affiliations:** 1 Department of Pharmacology and Therapeutics, University of Florida College of Medicine, Cancer and Genetics Research Complex, 2033 Mowry Rd., Gainesville FL 32610, USA; 2 J. Crayton Pruitt Family Department of Biomedical Engineering, University of Florida, Biomedical Sciences Building, Gainesville FL 32610, USA; 3 Cancer Diagnosis Program, Division of Cancer Treatment and Diagnosis, National Cancer Institute, Rockville MD 20852, USA; 4 Department of Radiation Oncology, University of Florida College of Medicine, 2000 SW Archer Road, Gainesville FL 32610, USA

**Keywords:** Angiogenesis, Angiopoietin-2, Anti-angiogenic therapy, Dorsal skinfold window chamber model, Vascular endothelial growth factor

## Abstract

**Background:**

Angiogenesis is an essential process during tumor development and growth. The murine dorsal skinfold window chamber model has been used for the study of both tumor microvasculature and other vascular diseases, including the study of anti-angiogenic agents in cancer therapy. Hyperspectral imaging of oxygen status of the microvasculature has not been widely used to evaluate response to inhibition of angiogenesis in early tumor cell induced vascular development. This study demonstrates the use of two different classes of anti-angiogenic agents, one targeting the Vascular Endothelial Growth Factor (VEGF) pathway involved with vessel sprouting and the other targeting the Angiopoietin/Tie2 pathway involved in vascular destabilization. Studies evaluated the tumor microvascular response to anti-angiogenic inhibitors in the highly angiogenic renal cell carcinoma induced angiogenesis model.

**Methods:**

Human renal cell carcinoma, Caki-2 cells, were implanted in the murine skinfold window chamber. Mice were treated with either VEGF pathway targeted small molecule inhibitor Sunitinib (100 mg/kg) or with an anti-Ang-2 monoclonal antibody (10 mg/kg) beginning the day of window chamber surgery and tumor cell implantation. Hyperspectral imaging of hemoglobin saturation was used to evaluate both the development and oxygenation of the tumor microvasculature. Tumor volume over time was also assessed over an 11-day period post surgery.

**Results:**

The window chamber model was useful to demonstrate the inhibition of angiogenesis using the VEGF pathway targeted agent Sunitinib. Results show impairment of tumor microvascular development, reduced oxygenation of tumor-associated vasculature and impairment of tumor volume growth compared to control. On the other hand, this model failed to demonstrate the anti-angiogenic effect of the Ang-2 targeted agent. Follow up experiments suggest that the initial surgery of the window chamber model may interfere with such an agent thus skewing the actual effects on angiogenesis.

**Conclusions:**

Results show that this model has great potential to evaluate anti-VEGF, or comparable, targeted agents; however the mere protocol of the window chamber model interferes with proper evaluation of Ang-2 targeted agents. The limitations of this *in vivo* model in evaluating the response of tumor vasculature to anti-angiogenic agents are discussed.

## Introduction

Angiogenesis, the formation of new blood vessels from pre-existing ones, is an essential process in tumor development and growth [1]. Angiogenesis can be separated into two key steps, (a) vascular destabilization mediated by the Angiopoietin/Tie2 axis and (b) endothelial cell activation heavily influenced by the Vascular Endothelial Growth Factor (VEGF) and its receptor VEGFR-2 [2,3]. The use of VEGF axis targeted anti-angiogenic agents has become a standard of care in many tumor settings in the last decade. However, their clinical efficacy has been limited [4,5]. Recently, a new class of anti-angiogenic agents targeting Angiopoetin-2 (Ang-2) has emerged in hopes to circumvent lack of response or resistance seen with VEGF targeted agents [6]. Currently many agents are being evaluated both in preclinical and clinical settings including targets not mentioned above [4,6].

The dorsal skinfold window chamber is commonly used to evaluate the microvasculature in various settings *in vivo*. The model involves surgical implantation of a titanium window onto exposed microvasculature on the dorsal skin of mice [7,8]. This method has been used to evaluate angiogenesis in a variety of processes such as endometriosis and tumor development; it was also used to evaluate the early phases of preclinical development of Bevacizumab in the 1990s [9-14]. Hyperspectral imaging of hemoglobin saturation has allowed for the evaluation of oxygenation and hypoxia, oxygen transport dynamics and characterization of the abnormal vascular physiology and acute oxygen fluctuations within the tumor microvasculature [15-18]. Tumor microvascular response to various therapies such as radiotherapy, vascular disrupting agents and sickled erythrocytes has also been evaluated using this imaging system [19-21]. Applications other than the tumor microvasculature have also been explored for example the characterization of arteriovenous malformation in hereditary hemorrhagic talengiectasia, the formation of spontaneous and induced microvascular thrombosis and occlusions and immune cell localization and tissue damage in particle based vaccines [18,22,23].

Anti-angiogenic agents are commonly evaluated *in vivo* using either matrigel plug or intradermal assays as well as histological assessments of the number and function of tumor associated vasculature [24]. To date, the murine dorsal skinfold window chamber model combined with hyperspectral imaging has not been widely utilized. This model allows for the evaluation of tumor response to anti-angiogenic agents and assesses not only the vascular density of the tumor but also its oxygenation status. Real time assessment of tumor vasculature at microvessel resolution has tremendous potential to answer several important questions regarding aspects of vascular response to anti-angiogenics such as oxygenation status of the vasculature. The current study evaluated both a VEGF and Ang-2 targeted approach on the induction of early human renal cell carcinoma cell induced angiogenesis. Limitations of this model in this particular setting quickly became apparent and are discussed here.

## Materials and methods

### Reagents

Mouse Ang-2 ELISA kit (MBS728992) was purchased from MyBioSource (San Diego, CA). MECA-32 (rat anti-mouse Cat# 120501) was purchased from BioLegend (San Diego, CA). NG2 (rabbit anti-mouse Cat# AB5320) was obtained from Millipore (Temecula, CA). AlexaFluor 488 (donkey anti-rabbit) and AlexaFluor 594 (donkey anti-rat) were purchased from Invitrogen (Grand Island, NY). VectaShield mounting medium with DAPI was purchased from Vector Labs Inc. (Burlingame, CA). Tissue-Tek OCT Compound was purchased from Sakura Finetek (Torrance, CA). 2-methylbutane was obtained from Thermo Fisher Scientific (Waltham, MA).

### Cell culture

The human clear cell renal cell carcinoma, Caki-2, cell line was originally received from Dr. Susan Knox (Stanford University). Caki-2 was grown in Dulbecco’s modified minimum essential medium (D-MEM, Invitrogen, Grand Island, NY) supplemented with 10% FBS (Invitrogen, Grand Island, NY), 1% penicillin-streptomycin (Invitrogen, Grand Island, NY), and 1% 200-mmol/L _L_-glutamine (Invitrogen, Grand Island, NY). Cells were kept at 37°C, 5% CO_2_.

### Drug preparation

Ketamine and xylazine were purchased from Webster Veterinary (Devens, MA) and prepared in sterile saline. The anti-Ang-2 monoclonal antibody was kindly provided by MedImmune, LLC. The stock solution (5 mg/ml) was diluted to the working dose (10 mg/kg) in sodium citrate buffer solution. Stock solutions were kept at -80°C and working concentrations at 4°C. The VEGFR small molecule inhibitor Sunitinib was obtained from LC Laboratories (Woburn, MA) and stored at -20°C. Working dose of Sunitinib was prepared fresh everyday by making stock and diluent buffers of citric acid monohydrate and sodium citrate dihydrate at pH 6.8 and 3.2 respectively. A 1:7 stock to diluent solution was made (~pH 3.3) and acidified to pH 1.0, Sunitinib was dissolved, and then the solution was adjusted to pH 3.5. Working concentration of Sunitinib was kept at room temperature.

### Window chamber surgery and tumor initiation

All *in vivo* procedures were conducted in agreement with a protocol approved by the University of Florida Institutional Animal Care and Use Committee. Dorsal skinflap window chamber surgeries were carried out as previously described by Moy and colleagues [7]. Briefly, female athymic nu/nu mice (Harlan Laboratories, Indianapolis, IN) were surgically implanted with a titanium window chamber on the dorsal skinflap. During the surgical procedure mice were anesthetized via intraperitoneal (IP) injection of ketamine (100 mg/kg) and xylazine (10 mg/kg). Human renal cell carcinoma, Caki-2, tumor was initiated in the window chamber during surgery by injecting cells (2 × 10^4^ in 10 μl volume) subcutaneously in the dorsal skinflap prior to placing a 12 mm diameter number 2 round glass cover slip (Erie Scientific, Portsmouth, NH) over the exposed skin. Post surgical procedure, animals were housed in an environmental chamber maintained at 33°C and 50% humidity with standard 12 hr light/dark cycles for the remainder of the study.

### Treatment of window chamber tumors

Mice were treated with Sunitinib (100 mg/kg) daily via oral gavage or with the anti-Ang-2 antibody (10 mg/kg) every 3 days via IP injection, starting the day of window chamber surgery/tumor initiation up to day 11 post-surgery when mice were euthanized. During the study, tumors were measured everyday using calipers and tumor volume (mm^3^) was calculated, assuming that the tumor volume was half of an ellipsoid, using the following equation: tumor volume = ½ [π/6 × d_1_ × d_2_ × height]. Statistical significance was determined using the Mann–Whitney *U*-Test at p < 0.05.

### Hyperspectral imaging

The spectral imaging system, image acquisition, and image processing methods have previously been described [7,15]. Briefly, window chamber tumors were imaged daily using a Zeiss AxioImager microscope (Carl Zeiss, Inc., Thornwood, NY) with 100-W tungsten halogen lamp, CCD camera thermoelectrically cooled to -20°C (DVC Co., Austin, TX; Model no.1412 AM-T2-FW) and C-mounted liquid crystal tunable filter (LCTF) (CRI Inc., Woburn, MA). Tuning of the LCTF and image acquisition with the CCD camera was automatically controlled with LabVIEW8 software (National Instruments Corp., Austin, TX). Vascular hemoglobin saturation measurements and images were created from the spectral image data, using Matlab software (The Mathworks Inc., Natick, MA). During imaging mice were placed on a heated platform and anesthetized with 1-2% isofluorane (Webster Veterinary, Devens, MA) in air.

### Immunohistochemistry

Eleven days post surgery mice were euthanized with euthasol (0.01 ml/g) (Webster Veterinary, Devens, MA), titanium window chambers were removed and tumors were fresh frozen in OCT and methylbutane. Tumors were sectioned at 5 μm thickness using Leica CM 3050S cryostat (Leica Microsystems Inc., Buffalo Grove, IL); sections were placed on superfrost plus gold slides (Thermo Fisher Scientific Inc., Waltham, MA) and kept at -80°C until immunohistochemical staining. Tissue sections were acetone fixed for 10 min, blocked in 2% normal horse serum, and incubated overnight at 4°C with MECA-32 and NG2 primary antibodies, at room temperature with secondary antibodies AlexaFluor 488 and 594 for 1 hr. Tissue sections were imaged with a Zeiss Axioplan 2 imaging microscope (Carl Zeiss, Inc., Thornwood, NY) with EXFO X-Cite 120 light source (Lumen Dynamics Group Inc., Ontario, Canada). Images were taken with a Retiga EXi Fast digital CCD camera (QImaging, British Columbia, Canada) and processed in OpenLab5 software (PerkinElmer Inc., Waltham, MA); Rhodamine for MECA-32/AlexaFluor594, FITC for NG-2/AlexaFluor488 and DAPI filters were used. Vessel counts were obtained by taking up to ten random fields/tumor at 20× objective, counting the number of vessels in each random field. The number of peri-endothelial cell covered vessels was counted in the tumor periphery for each tumor. Statistical significance between control and treated groups was determined using the Mann–Whitney *U*-Test at p < 0.05.

### Serum angiopoietin-2 levels

Blood from the tail vein was drawn on days 3 and 5 post surgery from mice (2 mice/group) that (1) did not have surgery (baseline), (2) that underwent surgery and (3) ones that underwent surgery and inoculated with tumor cells. About 100 μl of blood per mouse was collected and placed on ice for 2 hr to let the blood clot. Blood was then centrifuged for 15 min, 4°C at 1000 g. Serum was collected and stored at -80°C until analysis. Serum Ang-2 levels were calculated using a mouse-specific Ang-2 ELISA kit. Based on company recommendation, the manufacturer’s protocol was altered to load 30 μl of either standards or serum and 50 μl of 3.3 fold diluted conjugate in 0.9% NaCl per well and incubate for 2 hr at 37°C; manufacturers protocol was otherwise followed.

### Intradermal angiogenesis assay

All *in vivo* procedures were conducted in agreement with a protocol approved by the University of Florida Institutional Animal Care and Use Committee. Female athymic nu/nu mice were injected intradermally with 10^5^ Caki-2 cells in 10 μl volume at four sites on the ventral surface. Beginning the day prior to tumor cell injection, mice were treated with either daily oral gavage of Sunitinib (100 mg/kg) or IP injection of the anti-Ang-2 antibody (10 mg/kg) every 3 days up to six days post tumor cell inoculation. Mice were then euthanized, tumors measured via calipers and tumor volume (mm^3^) calculated, assuming the tumor volume to be an ellipsoid, using the following equation: tumor volume = π/6 × d_1_ × d_2_ × height. Skin flaps were then removed and vessels growing into tumor nodules were counted using a Leica MZ16F dissecting microscope with Leica KL 1500 LCD fiber optic illuminator (Leica Microsystems Inc., Buffalo Grove, IL) at 2.5x original magnification. Images were captured with a Retiga EXi Fast1394 digital CCD camera (QImaging, British Columbia, Canada) and OpenLab5 software (PerkinElmer Inc., Waltham, MA). Statistical significance between control and treated groups was determined using the Mann–Whitney *U*-Test at p < 0.05.

## Results

### Human renal cell carcinoma, Caki-2, growth in the window chamber

**Figure 1:**
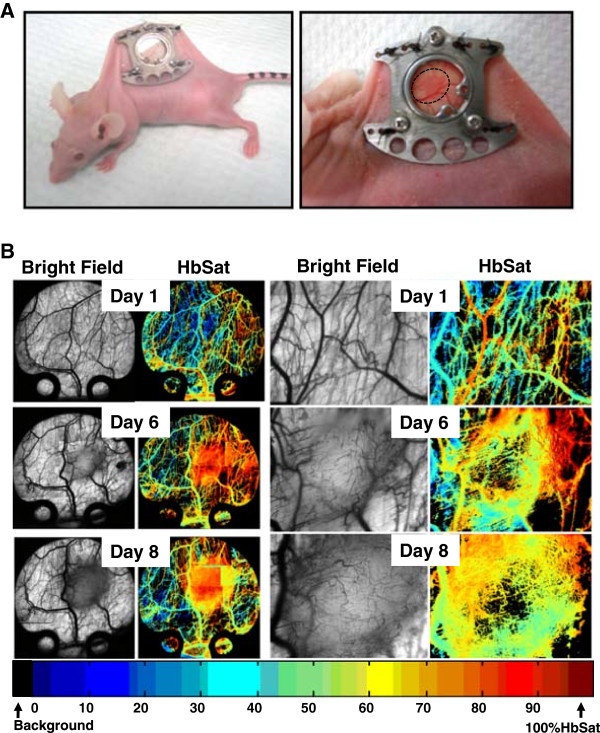
**Human renal cell carcinoma, Caki-2, tumor growth in the window chamber. (A)** Titanium chambers are surgically implanted in nude mice and tumor cells injected (2 × 10^4^) subcutaneously into the window. **(B)** Tumor cell induced microvascular development in the window chamber over time. Hyperspectral imaging of hemoglobin saturation provide oxygenation status of tumor microvasculature.

Renal cell carcinoma is a highly vascularized disease and anti-angiogenic agents are currently used as both first and second line treatments in patients. Caki-2 cells, a VHL mutant, highly vascular and aggressively growing cell line, were implanted into the window chamber (Figure 1A). Induction of tumor cell induced angiogenesis was seen at days 5–6 post tumor cell implantation at which point the tumors quickly expanded and became heavily vascularized with 50-80% hemoglobin saturation in the microvasculature at day 8 (Figure 1B).

### VEGF inhibition impedes tumor and vessel growth in Caki-2 tumors in the window chamber

**Figure 2:**
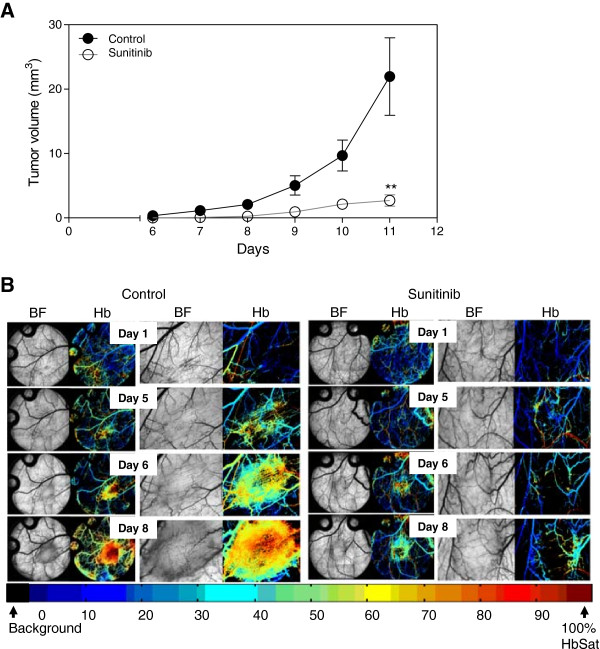
**VEGF inhibition in Caki-2 cell induced angiogenesis.** Mice bearing window chambers with Caki-2 tumors were treated with VEGF inhibitor (Sunitinib). **(A)** Tumor volume and **(B)** tumor microvascular response to Sunitinib was evaluated compared to control. Median + 90/10 percentile; control (n = 8), Sunitinib (n = 7) (combination of two independent experiments). **, p < 0.01, Mann–Whitney *U*-Test.

**Figure 3:**
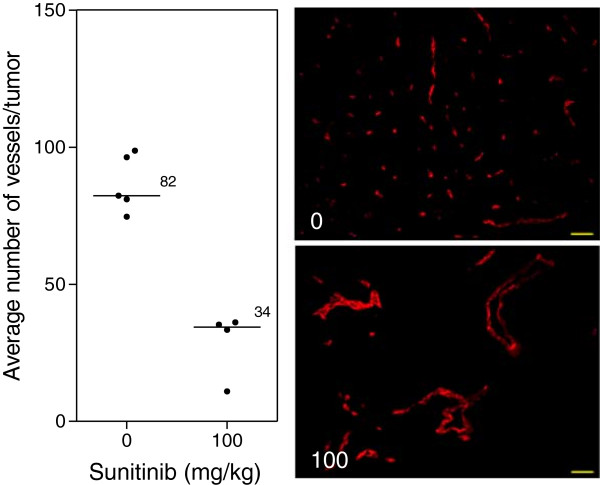
**Tumor vasculature after VEGF inhibition.** Immunohistochemical analysis of tumors at study endpoint. Line, median; *, p < 0.05; Mann–Whitney *U*-Test. Representative images of the median of each group. Red, MECA. Images taken with Zeiss Axioplan Imaging2 microscope with 20× objective; scale bar = 140 μm.

Caki-2 tumors grew rapidly and expanded their microvasculature greatly over time with increased oxygenation levels within the tumor mass. Treatment of mice with the VEGF inhibitor led to a significant 5.2-fold reduction in tumor volume (p < 0.01) (Figure 2A) as well as a dramatic impairment of tumor vasculature and oxygenation (Figure 2B). Immunohistochemical analysis of tumor vasculature at study endpoint revealed a 2.4-fold reduction in vessel number of the treated groups compared to control (p < 0.0001) (Figure 3).

### Ang-2 inhibition does not affect Caki-2 tumor growth in the window chamber

**Figure 4:**
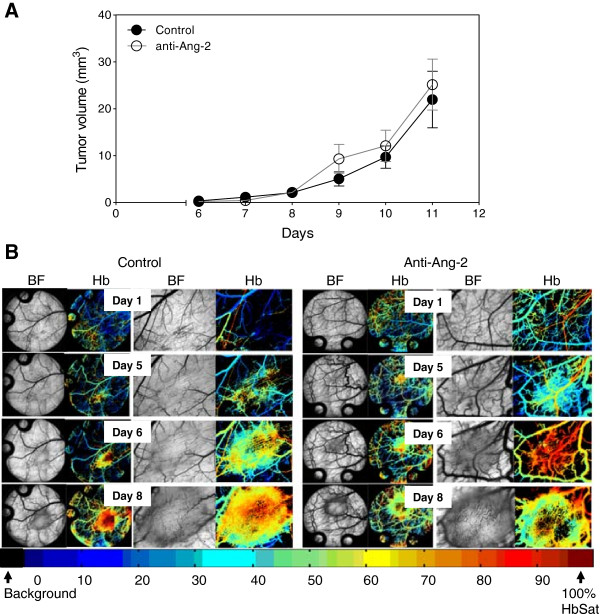
**Ang-2 inhibition in Caki-2 cell induced angiogenesis.** Mice bearing window chambers with Caki-2 tumors were treated with Ang-2 inhibitor (monoclonal antibody). **(A)** Tumor volume and **(B)** tumor microvascular response to the Ang-2 inhibitor was evaluated compared to control. Median + 90/10 percentile; control (n = 8), anti-Ang-2 (n = 7) (combination of two independent experiments).

**Figure 5:**
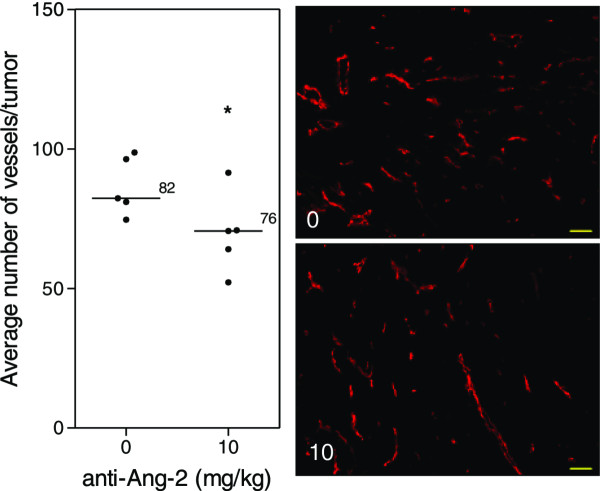
**Tumor vasculature after Ang-2 inhibition.** Immunohistochemical analysis of tumors at study endpoint. Line, median; *, p < 0.05; Mann–Whitney *U*-Test. Representative images of the median of each group. Red, MECA. Images taken with Zeiss Axioplan Imaging2 microscope with 20× objective; scale bar = 140 μm.

**Figure 6:**
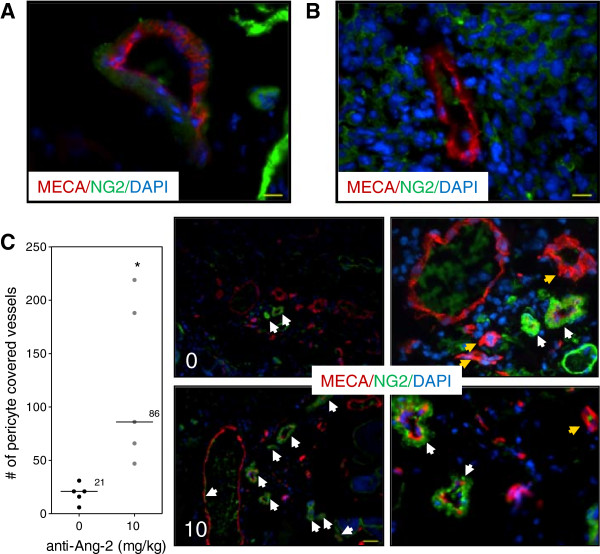
**Vascular structure after Ang-2 inhibition.** Immunohistochemical analysis of vascular structure at study endpoint. **(A)** Normal vasculature with pericyte coverage. **(B)** Tumor vasculature without pericyte coverage. Scale bar, 46 μm. **(C)** Ang-2 inhibition led to increased number of vessels that maintained pericyte coverage. Line, median; *, p < 0.05; Mann- Whitney *U*-Test. Representative images of each group. White arrows show pericyte covered vessels; yellow arrow shows vessels without pericyte coverage. Red, MECA (endothelium); green, NG2 (pericyte); blue, DAPI. Images taken with Zeiss Axioplan Imaging2 microscope with 20× objective; scale bar = 140 μm.

Treatment of mice with the Ang-2 inhibitor did not show impairment of tumor growth or vascular development (Figure 4). However, immunohistochemical analysis of tumor vasculature at study endpoint revealed a 1.1-fold reduction in the treated groups compared to control (p < 0.05) (Figure 5). Furthermore, analysis of the vascular structure revealed a 4-fold increase in the number of vessels that maintained pericyte coverage in the treated groups compared to control (p < 0.05) (Figure 6).

### VEGF and Ang-2 inhibition in the intradermal angiogenesis model

**Figure 7:**
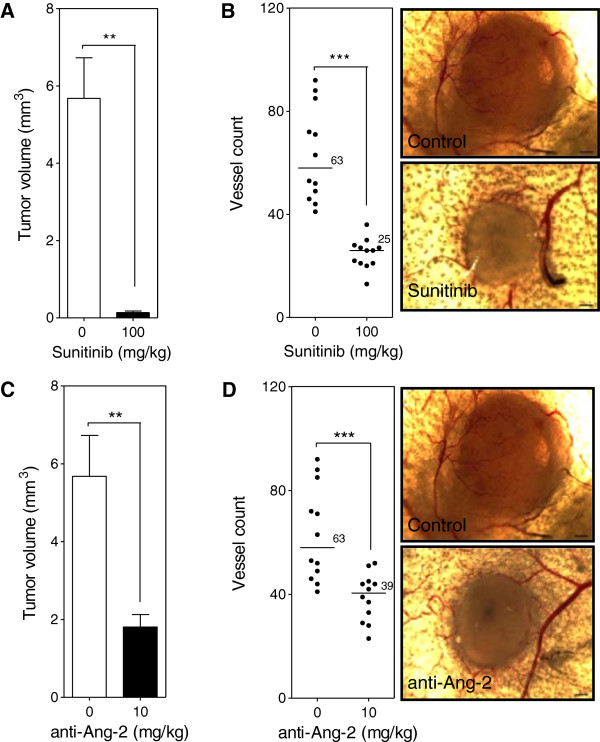
**Ang-2 and VEGF inhibition in the intradermal assay.** Mice were injected intradermally with Caki-2 renal cell carcinoma cells and treated with either the Ang-2 inhibitor (10 mg/kg) or Sunitinib (100 mg/kg) beginning the day prior to tumor cell inoculation. Tumor volume **(A, C)** and the number of tumor cell induced blood vessels **(B, D**) were determined at the end of a 7 day period. Bar, mean with SEM (n = 12); line, median (n = 12); **, p < 0.01; ***, p < 0.0001; Mann–Whitney *U*-Test.

To evaluate the inhibition of both the VEGF/VEGFR and Ang-2/Tie2 pathways in the absence of surgical installation of dorsal skinflold window chambers an intradermal assay was used. Inhibition of the VEGF/VEGFR pathway with Sunitinib (100 mg/kg) led to the reduction of both tumor and vessel growth by 43 (p < 0.001) (Figure 7A) and 2.5-fold (p < 0.0001) (Figure 7B) respectively compared to control. Treatment of mice with the anti-Ang-2 antibody (10 mg/kg) led to a reduction of both tumor and vessel growth by 3.1 (p < 0.001) (Figure 7C) and 1.6-fold (p < 0.0001) (Figure 7D) respectively compared to control.

### Surgery associated with the window chamber model led to an increase in serum Ang-2 levels

**Figure 8:**
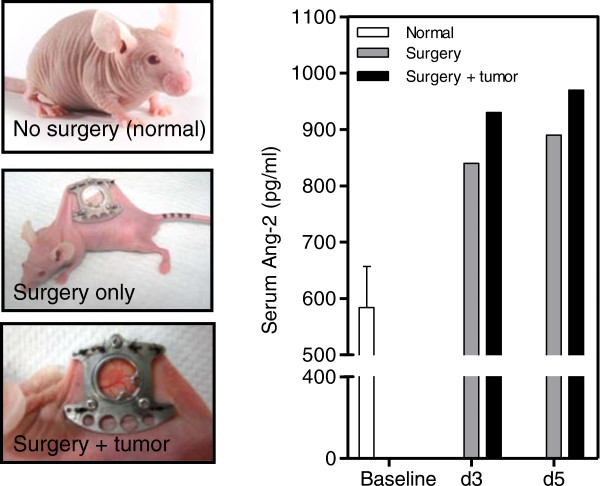
**Ang-2 serum levels in mice.** Levels of Ang-2 in the circulation were determined for mice that underwent window chamber surgery -/+tumor cell injection and compared to basal levels (no surgery).

The release of Ang-2 from endothelial cells as a wound healing response has been previously noted [25,26]. The circulating Ang-2 levels were determined in mice that underwent surgery. Results show that mice that underwent surgery had an increased level of Ang-2 in their circulation compared to control mice that did not receive surgery (Figure 8). Furthermore, the presence of tumor cells in mice that underwent surgery further increased the Ang-2 levels in the circulation compared to mice that only underwent surgery.

## Discussion

Vascular-targeted agents that inhibit the formation of new blood vessels from pre-existing ones have become a standard of care in several cancer settings over the last decade [4,27]. A cohort of patients do not respond or stop responding to treatment with the currently FDA approved anti-angiogenic agents. As a consequence new pathways in the angiogenic process have been exploited as therapeutic targets [4,6].

The murine dorsal skinfold window chamber model has been previously used to study and understand the microvasculature of not only tumors but other diseases as well [7-17,19,21-23]. Recently the use of hyperspectral imaging, to evaluate vascular oxygenation status through hemoglobin saturation absorption, allowed for more in depth evaluation of microvascular response to, among others, vascular-targeted agents and their effect on the microvasculature [20]. The dorsal skinfold window chamber model with high resolution hyperspectral imaging, to our knowledge, has not been widely used to evaluate anti-angiogenic therapy on the early initiation of angiogenesis and development of tumors. Ferrara and colleagues have used the window chamber model with basic imaging techniques to demonstrate the anti-angiogenic effects of Bevacizumab in preclinical development [11-14]. To date, there are no data in the literature using this model to evaluate Ang-2 targeted agents.

In the present study, two different classes of anti-angiogenic agents were evaluated. Sunitinib is a small molecule tyrosine kinase inhibitor that targets the VEGFR1-3 and PDGFR and has been FDA approved in 2006 as first line treatment in metastatic kidney cancer [28]. The anti-Ang-2 monoclonal antibody was used as the Ang-2 targeted agent. Results show that Caki-2 cells injected at 2×10^4^ cells initiate angiogenesis 5–6 days post tumor cell injection (Figure 1). When Caki-2 tumor bearing mice were treated with the VEGF inhibitor during an 11 day period the growth of the tumor was significantly inhibited by 5.2-fold compared to untreated tumors (Figure 2A). The development of tumor microvasculature of treated mice was significantly impaired with sparse and poorly oxygenated vessels (Figure 2B). Immunohistochemical analysis of tumors at endpoint further demonstrated the significantly impaired vasculature of treated mice with a 2.4-fold reduction in the number of vessels compared to control tumors (Figure 3). The results clearly show that the window chamber model and hyperspectral imaging can be a useful tool to evaluate agents targeting the VEGF pathway and the response of the tumor and microvasculature to such treatment. Results nicely demonstrate not only the inhibition of vascular development but also the poor oxygenation of the microvasculature that the tumor possesses.

On the other hand, the results show a different tumor response to the Ang-2 inhibitor. Mice treated with the antibody did not show any significant impairment of tumor growth (Figure 4A) compared to untreated tumors nor did it seem to have an effect on the vascular density or oxygenation of the vessels (Figure 4B). These results were rather puzzling at first but immunohistochemical analysis revealed a slight but significant reduction in the vessel number (Figure 5) as well as a highly significant 4-fold increase in pericyte covered vessels when tumors were treated with the Ang-2 inhibitor (Figure 6). Furthermore, previous results showed a significant anti-angiogenic effect of the Ang-2 inhibitor, which is the opposite response that is seen in the window chamber model [29]. Therefore, the intradermal assay was repeated with both the VEGF and Ang-2 inhibitors to closely mirror the experiment conducted in the window chamber model.

Results from the intradermal assay show that VEGF inhibition led to a significant 43- (Figure 7A) and 2.5-fold (Figure 7B) reduction in tumor volume and vessel number respectively compared to control correlating with the results seen in the window chamber model. Contrary to results seen with the Ang-2 inhibitor in the window chamber, the antibody led to a significant 3.1- (Figure 7C) and 1.6-fold (Figure 7D) reduction in tumor volume and vessel number compared to control. Similar tumor volume and vessel results with the VEGF targeted agent but opposing results with the Ang-2 targeted agent led to questions about the difference between the two models. In general, the sole difference between the intradermal assay and window chamber model is the initial surgery that is involved with the window chamber model. Review of the basic biology of the Ang-2 and VEGF axis in physiological response to injury led to the hypothesis that perhaps the surgery involved with the window chamber model leads to the rapid release of Ang-2 from damaged endothelial cells when the skinflap is cut to expose the vasculature on the skin that is spared. The angiopoietin axis is not only involved with the rapid response to vascular injury as a wound healing response but is also a pro-inflammatory factor [25,26,30-33]. The surgery to implant the window chamber could not only elicit a wound healing but also a pro-inflammatory response.To support this hypothesis serum samples from mice that have not received surgery, mice that received surgery and ones with surgery and tumor cell injection were evaluated at various time points after surgery. Results demonstrate that mice that received surgery had 1.4- and 1.5-fold higher Ang-2 levels in the serum compared to basal levels in mice that did not receive surgery at days 3 and 5 post surgery respectively (Figure 8). Furthermore, mice that received surgery and were injected with tumor cells had a slightly higher increase of 1.6-fold compared to baseline at days 3 and 5 post surgery (Figure 8).

In conclusion, the murine dorsal skinfold window chamber model is a valuable model to evaluate tumor microvasculature and vascular oxygenation using hyperspectral imaging. Cautions with this model should be taken when assessing the vascular response to therapeutic strategies. In this study two different classes of anti-angiogenic agents were evaluated. The Ang-2/Tie2 axis is important in vascular destabilization while the VEGF/VEGFR axis plays a role in endothelial cell activation to proliferate, migrate and form new vessels. While both axes are essential in physiological angiogenesis such as wound healing, they nevertheless have very different roles. Endothelial cells store Ang-2 in Weibel-Palade Bodies to be able to quickly respond to environmental changes such as vascular injury [34-36] while VEGF is essential in the formation of new vasculature, a later response in wound healing [37]. It is clear that the surgery involved with the window chamber model upsets the normal balance of Ang-2 in the microenvironment leading to skewed results to Ang-2 inhibition. The window chamber model, however, is a great tool to evaluate the inhibition of endothelial cell activation and could be utilized to rapidly and effectively evaluate anti-angiogenic agents in preclinical studies.

## Competing interests

The authors declare that they have no competing interests.

## Authors’ contributions

NMB helped with the design of the study, carried out all experiment and drafted the manuscript. JAL participated in window chamber surgeries, hyperspectral imaging and blood collection and edited the manuscript. BSS participated in the design of the study, helped with hyperspectral imaging and edited the manuscript. DWS was the principal investigator, helped design this study and edited the manuscript. All authors read and approved the final manuscript.
